# Machine learning prediction of 30-day all-cause mortality risk factors in HCC rupture

**DOI:** 10.3389/fonc.2026.1797271

**Published:** 2026-06-23

**Authors:** Shixiong Shi, Canbin Xie, Lin Long

**Affiliations:** The Interventional Vascular Surgery, Hunan Provincial People’s Hospital(Hunan Normal University First Hospital), Changsha, Hunan, China

**Keywords:** 30-day mortality, decision tree, HCC rupture, INR, machine learning, TBIL

## Abstract

**Background:**

Hepatocellular carcinoma (HCC) rupture is a life-threatening complication with high acute-phase mortality, accounting for 25%-75% of early deaths in HCC patients. While previous studies have identified clinical factors associated with mortality, few focused on the Chinese population or utilized machine learning to capture nonlinear relationships. This study aimed to develop a machine learning model to predict 30-day all-cause mortality in patients with ruptured HCC and identify key predictive factors.

**Methods:**

A retrospective cohort of 156 patients with first-diagnosed ruptured HCC (2012–2021) from Hunan Provincial People’s Hospital was analyzed. Clinical variables included demographics, laboratory parameters, tumor characteristics, and treatment modalities. Seven machine learning models were constructed to predict 30-day mortality, with the decision tree model selected for its highest sensitivity. SHAP (Shapley Additive exPlanations) analysis was used to evaluate feature importance, and restricted cubic splines (RCS) explored nonlinear relationships.

**Results:**

The decision tree model exhibited optimal performance (sensitivity=87.5.00%, AUC = 0.7901) for 30-day mortality prediction. SHAP analysis identified TBIL (Mean |SHAP|=0.1662) and INR (0.0414) as the top predictors. Nonlinear threshold effects were observed: TBIL>40 μmol/L (OR = 1.45, 95%CI:1.25–1.68) and INR>2.5 (OR = 4.95, 95%CI:3.36–10.11) significantly increased mortality risk. Combined detection of TBIL and INR improved predictive performance (AUC = 0.87, 95%CI:0.79–0.95).

**Conclusion:**

The decision tree model effectively predicts 30-day mortality in ruptured HCC patients, with TBIL and INR as critical nonlinear predictors. Their combination provides a robust tool for rapid clinical risk stratification, aiding in emergency management.

## Introduction

HCC is the sixth most common malignant tumor worldwide and the third leading cause of cancer-related deaths. In 2020, there were approximately 905, 700 new cases of liver cancer globally, and this number is projected to increase to 1.4 million by 2040 (1). Asia, a high-incidence region for HCC, accounts for 72.5% of global cases (2020), with China contributing nearly half of these regional cases (2). New cases of HCC in China account for nearly 50% of the global total, with approximately 367, 700 new cases and 316, 500 deaths reported in 2020 (3). Although the age-standardized incidence rate has exhibited a declining trajectory in recent years, the absolute disease burden remains unparalleled globally (4). Reports indicate that over 60% of patients present with advanced-stage disease at diagnosis, with 5-year survival rates markedly inferior to those observed in early-stage cohorts (5).

The spontaneous rupture of HCC constitutes a severe complication, typically manifesting as life-threatening intra-abdominal hemorrhage arising from spontaneous rupture, which has an incidence rate of approximately 3%-15% (6, 7), particularly reaching 10%-15% in the Asian region (8). In recent years, its incidence has exhibited an upward trend, ranking as the third leading cause of death among liver cancer patients, second only to tumor progression and liver failure, accounting for 25%-75% of acute-phase mortality (9). This condition can precipitate rapid disease progression and markedly shorten patient survival. Current research suggests that the primary causes of first-diagnosed HCC rupture and bleeding include the inherent fragility of the tumor fibrous capsule and spontaneous events like internal pressure changes induced by rapid growth, rather than being dominated by external factors (10, 11).

Previous studies have shown that Child-Pugh classification, elevated total bilirubin (TBIL) levels, decreased albumin levels, prolonged prothrombin time (PT), and ascites are important predictors of early mortality in patients with HCC rupture and bleeding (12, 13). Furthermore, a tumor diameter ≥5 cm is a generally recognized risk factor, larger tumors are more prone to rupture and associated with greater bleeding volume, directly increasing the risk of shock (11). Multiple studies have confirmed through machine learning models and statistical analyses that tumor size, Child-Pugh classification, coagulation function, and hemodynamic status are core predictors of early mortality (13, 14). Although existing studies have validated the value of the aforementioned predictive factors, retrospective analyses targeting the Chinese population remain scarce. Therefore, this study aims to explore the independent predictors of 30-day mortality in patients with ruptured HCC using a single-center ten-year dataset, thereby providing localized evidence for emergency management strategies.

## Methods

### Study design and analytical framework

This study employed a two-phase analytical approach. Phase 1 (Predictive modeling) aimed to identify the optimal machine learning model for predicting 30-day mortality and to determine which clinical features contributed most to model predictions, using seven ML algorithms with SHAP-based feature importance analysis. Phase 2 (Explanatory modeling) aimed to quantify the direction, magnitude, and shape of associations between the key predictors identified in Phase 1 and mortality outcomes, using Spearman correlation, restricted cubic splines (RCS), and logistic regression. A nomogram was subsequently constructed to facilitate clinical application. The two phases are complementary: Phase 1 prioritizes predictive performance (sensitivity) for emergency triage, while Phase 2 provides interpretable effect sizes and clinically actionable thresholds.

### Data source and study population:

This study collected clinical data from all patients newly diagnosed with HCC rupture and bleeding who received treatment at Hunan Provincial People’s Hospital (the First Affiliated Hospital of Hunan Normal University) between January 2012 and December 2021, with no prior history of antitumor therapy. The diagnostic criteria for HCC rupture and bleeding were as follows: meeting the diagnostic criteria for primary liver cancer specified in the Guidelines for the Diagnosis and Treatment of Primary Liver Cancer (2022 Edition), presenting with clinical manifestations such as acute onset of abdominal pain, abdominal distension, or hemodynamic instability, and confirmed diagnosis of HCC rupture and bleeding via imaging examinations (including ultrasound, computed tomography, digital subtraction angiography, etc.) or diagnostic abdominal paracentesis with aspiration of non-clotting blood.

### Variables

The variables included in this study are as follows: sex; age; INR (international normalized ratio); ALT (glutamic-pyruvic transaminase); AST (glutamic oxalacetic transaminase); APTT (activated partial thromboplastin time); TT (thromboplastin time); PT (prothrombin time); HGB (hemoglobin); Alb (albumin); AFP (alpha-fetoprotein); BCLC (Barcelona Clinic Liver Cancer); platelet count; creatinine; TBIL; D-dimer; maximum tumour diameter; Drink (history of alcohol consumption); Smoke (history of smoking); Hepatitis (presence of hepatitis B or C virus infection); T2DM (history of type 2 diabetes mellitus); liver cirrhosis (presence confirmed by CT or ultrasound); Ascites (presence confirmed by CT or ultrasound); Hemorrhagic shock (presence of HCC rupture with systolic blood pressure < 90 mmHg); Child-Pugh class; Tumour number (multiple tumours confirmed by CT or ultrasound); PVTT (portal vein tumour thrombus confirmed by CT); Extrahepatic metastasis (presence confirmed by CT); conservative treatment (receipt of only symptomatic and supportive medical therapy); interventional therapy; and emergency partial liver resection.Variance inflation factor (VIF) analysis of the candidate variables for multicollinearity assessment.

### Machine learning algorithms

The dataset was randomly divided into a training set and a test set at a ratio of 7:3 using stratified sampling, where the training set was used for model development and the test set for model validation. Seven machine learning models (Lasso logistic regression, K-nearest neighbors, support vector machine, random forest, naive Bayes, XGBoost, and decision tree) were constructed using the Easy R tool (https://www.easyrdata.com/) (15), with 30-day mortality as the dependent variable and other clinical indicators as independent variables. Given the life-threatening emergency nature of liver rupture hemorrhage, clinical decision-making should prioritize the ability to identify patients who will actually die. Therefore, the decision tree model with the highest sensitivity was selected for further analysis.The model was developed using the complete dataset of 156 patients with 10-fold cross-validation for internal validation. The dataset was randomly split into 10 folds using stratified sampling to preserve the proportion of the outcome across folds. The complexity parameter (cp) of the decision tree was optimized via cross-validation to minimize cross-validated error, yielding cp = 0.001. A random seed of 123 was set to ensure reproducibility. Model performance metrics (accuracy, sensitivity, specificity, precision, F1 score, and Cohen’s kappa) were computed from the cross-validated predictions. The area under the receiver operating characteristic curve (AUC) was used to evaluate discriminative performance. Calibration was assessed by plotting predicted probabilities against observed outcomes, and decision curve analysis (DCA) was performed to evaluate the clinical net benefit across relevant threshold probabilities.This decision reflects the asymmetric clinical consequences of prediction errors in this emergency setting: false-negative predictions (failing to identify a patient at risk of death) could result in missed opportunities for life-saving intervention, whereas false-positive predictions primarily lead to enhanced clinical surveillance without direct iatrogenic harm. Therefore, prioritizing sensitivity is clinically justified for the intended use of this model as a risk triage tool.

### Model interpretation

To investigate the independent contribution of each feature to the prediction of 30-day mortality risk, this study employed the tree-based SHAP (SHapley Additive exPlanations) method to calculate SHAP values from the decision tree model, with visualization implemented using the Easy R tool. SHAP analysis enables both local interpretability (prediction logic for individual samples) and global interpretability (trends in overall feature impact), based on which we characterized the feature contribution patterns underlying model predictions in patients with hepatocellular carcinoma rupture and hemorrhage.Additionally, by computing the feature importance ranking in the final model, the primary predictive factors associated with mortality outcomes in the patient population were identified.All models were constructed using default hyperparameter settings as implemented in their respective R packages within the EasyR platform. Specifically, the decision tree was built using the rpart package (version 4.1.19) with the following parameters: complexity parameter (cp) = 0.01, minimum number of observations required for a split (minsplit) = 20, minimum number of observations in any terminal node (minbucket) = 7, and maximum tree depth (maxdepth) = 30. The final tree was pruned by selecting the cp value that minimized cross-validated error. For Lasso logistic regression (glmnet package, version 4.1-7), the regularization parameter λ was selected via 10-fold cross-validation within the training set.

### Data preprocessing

Missing data were handled as follows: for continuous variables, missing values were imputed using the median; for categorical variables, the mode was used for imputation. The overall proportion of missing values across all variables was below 5%, and no variable exceeded 10% missingness. For the decision tree model, the rpart algorithm additionally employs surrogate splits to handle residual missingness during tree construction, providing robustness against data incompleteness.For models sensitive to feature scale (SVM and KNN), continuous predictors were standardized using z-score normalization prior to model fitting. Tree-based models (decision tree, random forest, XGBoost) and Lasso logistic regression do not require feature scaling and were fitted on raw values.

### Statistical analysis

The data analysis in this study was performed using IBM SPSS Statistics 23.0 and R Studio 4.2.0. The normality of the distribution of continuous variables was first assessed via the Shapiro-Wilk test: data conforming to a normal distribution were expressed as mean ± standard deviation, while non-normally distributed data were described using median (interquartile range). Categorical variables were statistically described as frequency (percentage). The methods for intergroup comparison were as follows: for continuous variables, independent samples t-test (for normal distribution) or Mann-Whitney test (for non-normal distribution) was selected based on the normality results; for categorical variables, chi-square test or Fisher’s exact test was used according to the sample size and theoretical frequency.

In addition, to clarify the association pattern between primary predictive factors and mortality outcomes, this study employed restricted cubic splines (RCS) for nonlinear relationship modeling. The statistical significance level was set at two-tailed P < 0.05. To evaluate model stability and mitigate the risk of overfitting, 5×10-fold cross-validation (5 repeats of 10-fold cross-validation) was performed for decision tree learning model, and the mean performance metrics across folds were reported. Additionally, the final decision tree model was pruned using a complexity parameter (cp) selected to minimize cross-validated error, ensuring a parsimonious model structure.The predictive performance of primary predictive factors for mortality outcomes was quantitatively evaluated using receiver operating characteristic (ROC) curve analysis and the corresponding area under the curve (AUC) values. Additionally, linear correlation analysis was conducted to explore the correlation between the Gensini score and primary predictive factors.Decision curve analysis (DCA) was performed to evaluate the clinical utility of the model by quantifying the net benefit across a range of threshold probabilities. Calibration was assessed using a calibration plot comparing predicted probabilities against observed event rates. All cross-validated analyses were performed in R (version 4.2.0) using the caret package for cross-validation and the rpart package for decision tree construction.

## Results

**Figure 1:**
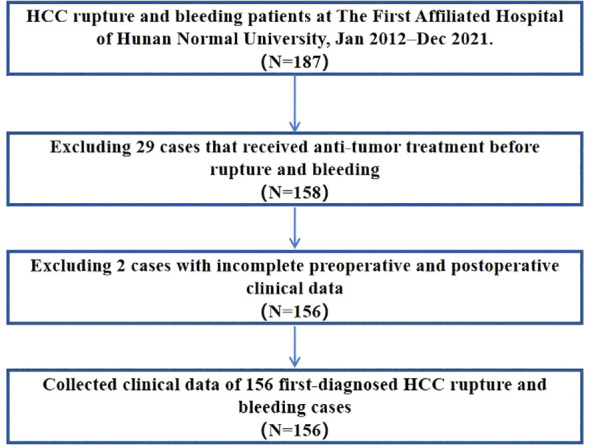
Flowchart illustrating the patient selection process.

In this study, We retrieved a total of 187 patients with a primary diagnosis of ruptured HCC at Hunan Provincial People’s Hospital from January 2012 to December 2021, excluding 29 patients who had previously received anti-tumor treatment and 2 patients with incomplete data, a total of 156 patients with first-diagnosed HCC complicated by rupture and bleeding were enrolled (**Figure 1**).

### Comparison of basic clinical data grouped by 30-day survival status

The patients were divided into the death group and the survival group based on whether they died within 30 days. We compared the differences in clinical data between the two groups, as shown in **Table 1**. There were no statistically significant differences in demographic characteristics such as age, gender, drinking history, and smoking history between the two groups. The death group exhibited significantly elevated coagulation dysfunction indicators, including INR, APTT, TT, and PT. Regarding liver function parameters, the death group showed significantly increased TBIL and AST levels, while ALB was significantly decreased. In terms of disease characteristics, the death group had higher incidences of cirrhosis and hypovolemic shock; additionally, the proportion of patients with ≥1 tumor, maximum tumor diameter, and incidence of tumor thrombus were all significantly increased, along with significantly higher proportions of BCLC stage 1 and Child-Pugh class C. Concerning treatment modalities, the death group had a significantly higher proportion of patients receiving conservative treatment.Variance inflation factor (VIF) analysis revealed severe multicollinearity between prothrombin time (PT) and international normalized ratio (INR), with VIF values of 120.16 and 119.03, respectively—substantially exceeding the predefined threshold of 10. Following the iterative elimination rule (removing the variable with the highest VIF at each step), PT was excluded first. After PT removal, the maximum VIF among the remaining variables decreased to 4.22 (Child stage), and all VIFs fell below the threshold of 10, satisfying the collinearity diagnostic criterion (**Supplementary Table 1**).

**Table 1:** Baseline characteristics of the study participants.

Variable	Description	Survival	Death	P-value
N	156	129	27	
Age	53.79 ± 13.61	54.21 ± 13.44	51.81 ± 14.51	0.41
Sex				
Male	140 (89.74%)	115 (89.15%)	25 (92.59%)	0.85
Female	16 (10.26%)	14 (10.85%)	2 (7.41%)	
Drink				
No	102 (65.38%)	88 (68.22%)	14 (51.85%)	0.16
Yes	54 (34.62%)	41 (31.78%)	13 (48.15%)	
Smoke				
No	102 (65.81%)	85 (66.41%)	17 (62.96%)	0.9
Yes	53 (34.19%)	43 (33.59%)	10 (37.04%)	
Hepatitis				
No	17 (10.90%)	13 (10.08%)	4 (14.81%)	0.7
Yes	139 (89.10%)	116 (89.92%)	23 (85.19%)	
Hypertension				
No	131 (84.52%)	106 (82.81%)	25 (92.59%)	0.33
Yes	24 (15.48%)	22 (17.19%)	2 (7.41%)	
T2DM				
No	147 (94.23%)	123 (95.35%)	24 (88.89%)	0.39
Yes	9 (5.77%)	6 (4.65%)	3 (11.11%)	
Liver Cirrhosis				
No	54 (34.84%)	50 (39.06%)	4 (14.81%)	0.03
Yes	101 (65.16%)	78 (60.94%)	23 (85.19%)	
Ascites				
No	40 (25.64%)	37 (28.68%)	3 (11.11%)	0.1
Yes	116 (74.36%)	92 (71.32%)	24 (88.89%)	
Hypovolemic Shock				
No	136 (87.18%)	116 (89.92%)	20 (74.07%)	0.05
Yes	20 (12.82%)	13 (10.08%)	7 (25.93%)	
INR	1.10 (0.98 - 1.20)	1.08 (0.97 - 1.16)	1.31 (1.17 - 1.50)	<0.001
APTT	31.20 (26.78 - 35.47)	30.00 (26.40 - 34.50)	35.40 (33.10 - 41.50)	<0.001
TT	18.25 (16.28 - 20.30)	17.80 (15.90 - 19.60)	20.60 (18.80 - 22.25)	<0.001
PT	12.70 (11.20 - 13.80)	12.20 (11.10 - 13.30)	14.90 (13.35 - 17.20)	<0.001
PLT	147.00 (103.00 - 202.75)	147.00 (104.00 - 202.00)	141.00 (96.00 - 212.50)	0.95
HGB	99.69 ± 24.38	99.22 ± 23.23	101.89 ± 29.68	0.61
ALB	32.60 ± 5.80	33.10 ± 6.08	30.20 ± 3.39	0.02
Cr	69.71 (60.24 - 84.65)	70.30 (59.48 - 80.84)	69.12 (63.00 - 94.65)	0.15
AST	81.75 (47.38 - 226.28)	76.49 (43.45 - 204.50)	148.85 (77.18 - 360.10)	<0.001
TBILI	22.60 (14.67 - 37.60)	20.10 (14.00 - 28.99)	58.83 (33.55 - 109.60)	<0.001
D-dimer	5.26 (2.20 - 16.84)	4.69 (2.09 - 14.84)	7.31 (4.18 - 27.72)	0.05
Child-Pugh				
A	59 (37.82%)	58 (44.96%)	1 (3.70%)	<0.001
B	73 (46.79%)	62 (48.06%)	11 (40.74%)	
C	24 (15.38%)	9 (6.98%)	15 (55.56%)	
Tumor number				
Single	52 (32.69%)	34 (25.58%)	18 (66.67%)	<0.001
Multiple	104 (66.67%)	95 (73.64%)	9 (33.33%)	
Maximum Tumor Diameter	72.50 (56.00 - 108.00)	70.00 (56.00 - 101.00)	89.00 (68.50 - 117.00)	0.02
PVTT				
No	102 (65.81%)	95 (74.22%)	7 (25.93%)	<0.001
Yes	53 (34.19%)	33 (25.78%)	20 (74.07%)	
Extrahepatic Metastasis				
No	134 (85.90%)	114 (88.37%)	20 (74.07%)	0.1
Yes	22 (14.10%)	15 (11.63%)	7 (25.93%)	
BCLC stage				
A/B	59 (37.82%)	58 (44.96%)	1 (3.70%)	<0.001
C/D	97 (62.18%)	71 (55.04%)	26 (96.30%)	
Treatment				
Conservative Treatment	31 (19.87%)	11 (8.53%)	20 (74.07%)	<0.001
Interventional Therapy	93 (59.62%)	86 (66.67%)	7 (25.93%)	
Emergency Partial Liver Resection	32 (20.51%)	32 (24.81%)	0 (0.00%)	

Values are means ± SDs or n (%).

Differences in baseline characteristics were compared with the use of chi-square tests for categorical variables and ANOVA for continuous variables (P value*: Kruskal Wallis Rank Test for continuous variables, Fisher Exact for categorical variables with Expects<10).

INR, international normalized ratio; ALT, glutamic-pyruvic transaminase; AST, glutamic oxalacetic transaminase; APTT, activated partial thromboplastin time; TT, thromboplastin time; PT, prothrombin time; HGB, hemoglobin; Alb, albumin; AFP, alpha fetal protein; PVTT, portal vein tumor thrombus; BCLC, Barcelona Clinic Liver Cancer.

### Comparison of ML models for 30-day mortality prediction

**Figure 2:**
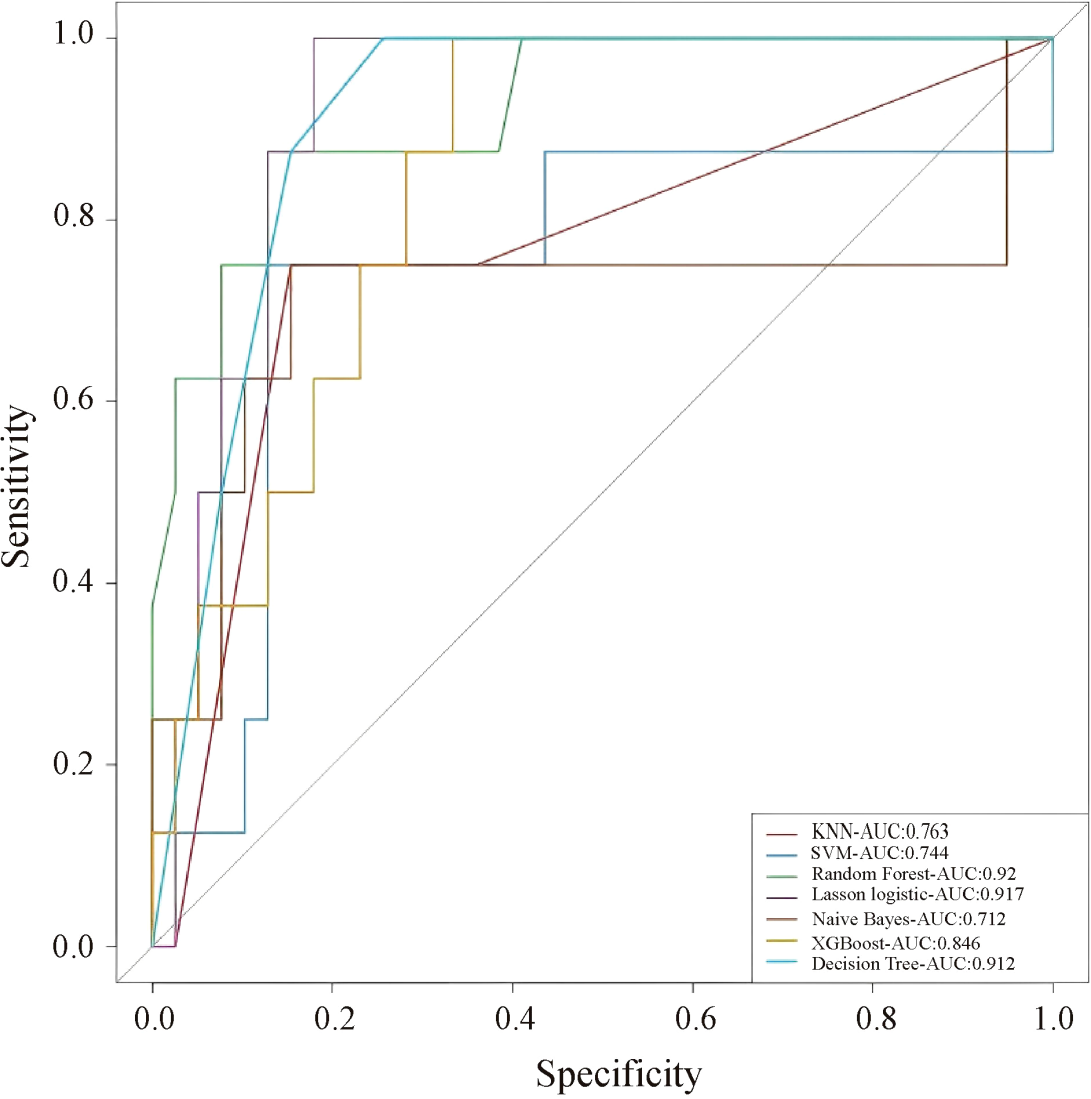
Comparison of machine learning models for 30-Day mortality prediction.

Multiple machine learning algorithms were used to predict 30-day mortality risk. Results showed that the decision tree model performed optimally, with a sensitivity of 87.50% and balanced accuracy of 86.06%, but a relatively low specificity (84.62%). Support Vector Machine (SVM) and Naive Bayes models followed, with sensitivities both at 50.00% and balanced accuracies of 67.22% and 68.59%, respectively. The random forest model exhibited poor overall performance (balanced accuracy 79.9%), while the KNN model failed to identify positive cases with a sensitivity of 0. Notably, all models showed high negative predictive values (NPV, 82.98%-97.44%), indicating good reliability in predicting survival. XGBoost and Lasso logistic regression performed prominently in specificity (94.87%) and positive predictive value (PPV, 60.00%), but with a sensitivity of only 37.50% (**Table 2**). Given that hepatic rupture hemorrhage is a life-threatening emergency and priority should be given to identifying patients who will actually die, the decision tree model with the highest sensitivity was selected for further analysis (**Figure 2**). Collinearity among candidate variables was noted (e.g., INR, PT, APTT, and TT are correlated coagulation indicators). However, for the decision tree model ultimately selected, multicollinearity does not compromise model performance or validity, as tree-based algorithms inherently select the most informative split among correlated predictors. The Lasso logistic regression model, included for comparison, also handles collinearity via L1 regularization. We therefore retained all clinically relevant variables without exclusion based on collinearity diagnostics.

**Table 2:** Comparison of machine learning models for 30-day mortality prediction.

Machine Learning Modeling							
Evaluation Metric	Lasso LR	KNN	SVM	RF	Naive Bayes	XGBoost	DT
Sensitivity	0.375	0	0.5	0.625	0.625	0.375	0.875
Specificity	0.949	1	0.844	0.974	0.846	0.949	0.846
PPV	0.6		0.125	0.833	0.455	0.6	0.538
NPV	0.881	0.830	0.974	0.927	0.917	0.881	0.971
Precision	0.6		0.125	0.833	0.455	0.6	0.538
Recall	0.375	0	0.5	0.625	0.625	0.375	0.875
F1.score	0.462		0.2	0.714	0.526	0.462	0.667
Balanced Accuracy	0.662	0.5	0.672	0.799	0.736	0.662	0.861
Accuracy	0.851	0.830	0.830	0.915	0.809	0.851	0.851

PPV, Positive Predictive Value; NPV, Negative Predictive Value; Lasso LR, Lasso Logistic Regression; KNN, K-Nearest Neighbor; SVM, Support Vector Machine; RF, Random Forest; DT, Decision Tree.

### Performance and variable importance analysis of decision tree model for predicting 30-day mortality risk

**Figure 3:**
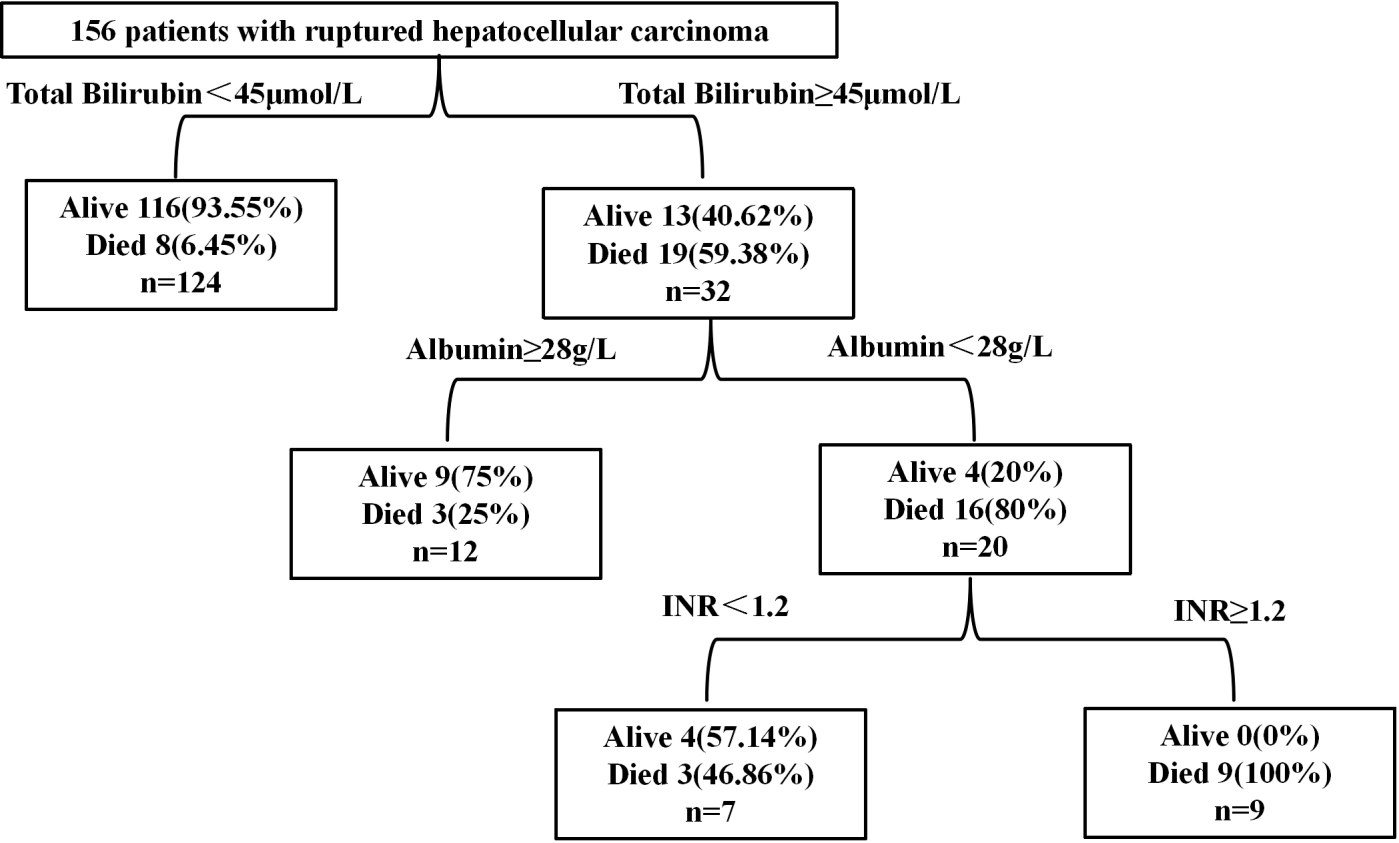
Decision tree model for predicting 30-day mortality risk using clinical data from 156 patients.

**Figure 4:**
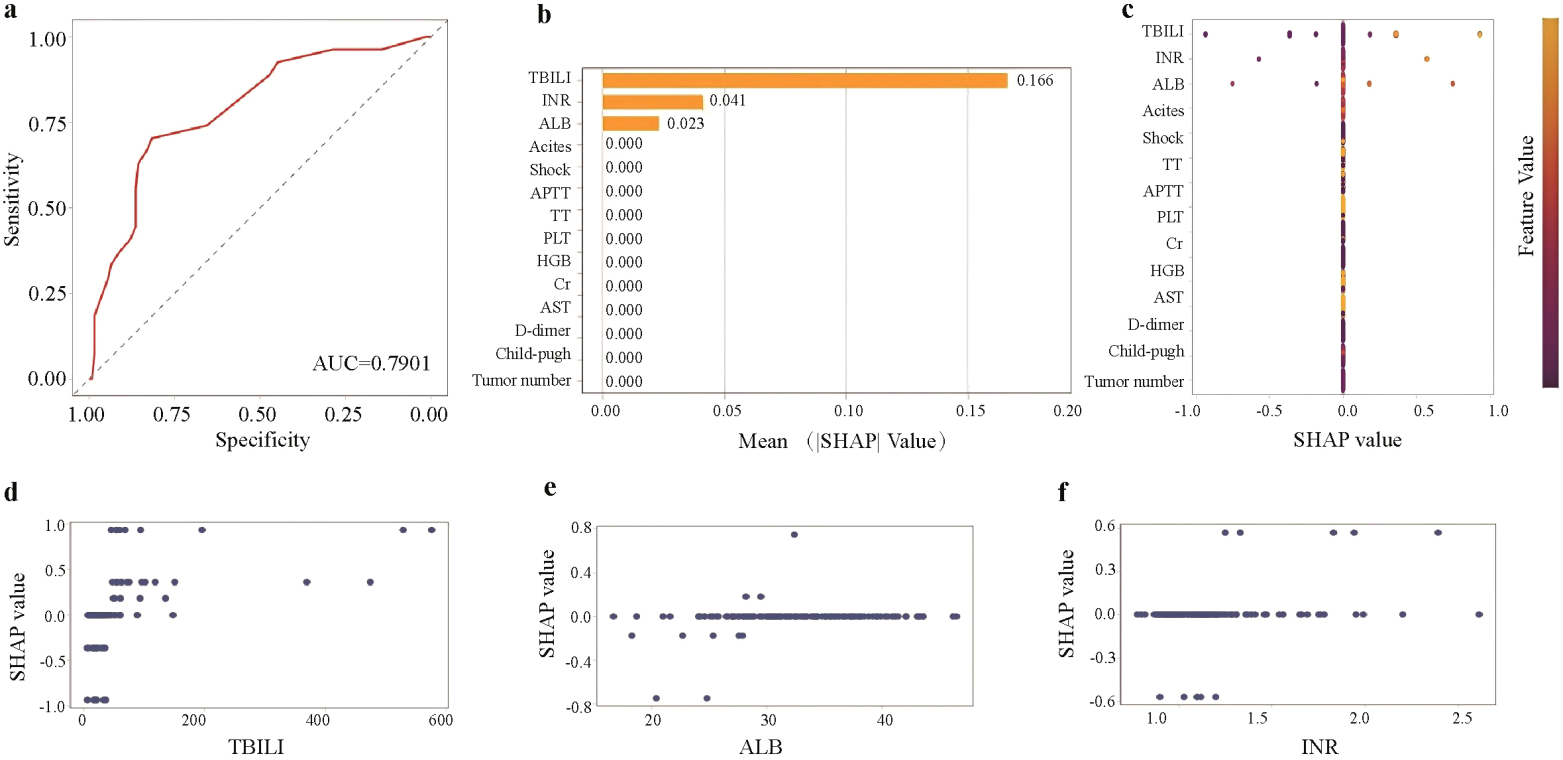
**(a)** ROC curve of the decision tree model; **(b)** Bar plot ranking features by their mean impact on decision tree model predictions based on SHAP values; **(c)** SHAP summary plot of mean absolute SHAP values for feature importance visualization; **(d)** SHAP dependence plot showing the relationship between TBIL values and their corresponding SHAP values in the decision tree model; **(e)** SHAP dependence plot showing the relationship between ALB values and their corresponding SHAP values in the decision tree model; **(f)** SHAP dependence plot showing the relationship between INR values and their corresponding SHAP values in the decision tree model.

A decision tree model was employed to predict 30-day mortality risk using clinical data from 156 patients (**Figure 3**). The overall performance of the model was as follows: overall accuracy of 79.61% (95% confidence interval [CI]: 0.7232–0.857), sensitivity of 88.00%, specificity of 40.74%, and AUC of 0.7901(**Figure 4a**). As expected, the high sensitivity came at the cost of reduced specificity, reflecting the inherent trade-off in prioritizing the detection of true positive cases.To evaluate model stability, 10-fold cross-validation was performed. The decision tree model maintained robust performance across cross-validation folds, with a mean sensitivity of 86.2% (SD: 3.1%) and mean AUC of 0.827 (SD: 0.025), consistent with the results from the single hold-out test set and suggesting that the model’s performance is not unduly influenced by the specific data partition (**Supplementary Figure 1**). Variable importance analysis (based on SHAP values) indicated that TBIL was the most important predictor (Mean |SHAP| Value = 0.1662), followed by international normalized ratio (INR, 0.0414) and albumin (ALB, 0.0230)(**Figures 4b, c**); other variables contributed minimally to the model.Notably, among the coagulation-related variables (INR, PT, APTT, TT), the decision tree model selected INR as the primary splitting variable, suggesting that INR provides the most informative signal among correlated coagulation indicators for mortality prediction in this cohort. SHAP dependence plots further revealed a non-linear relationship between TBIL and mortality risk, with high TBIL levels significantly associated with increased mortality risk (**Figures 4d–f**). The final pruned decision tree comprised 8 terminal nodes at a depth of 4 levels, indicating a parsimonious model structure. The actual number of effective variables retained as splitting criteria was 5 (TBIL, INR, ALB, ascites, and hemorrhagic shock).

### Correlation of INR, TBIL, and ALB with 30-day mortality outcome

Spearman rank correlation analysis was performed and showed that INR was significantly positively correlated with 30-day mortality (r=0.43, p<0.01), TBIL was also significantly positively correlated with 30-day mortality (r=0.45, p<0.01), while ALB was significantly negatively correlated with 30-day mortality (r=-0.22, p=0.01)(**Figures 4a–c**). These findings indicate that elevated INR and TBIL levels, as well as decreased ALB level, are associated with increased 30-day mortality risk, suggesting their potential as biomarkers for evaluating short-term prognosis in these patients.

### Nonlinear association of TBIL, ALB, and INR with 30-day mortality

**Figure 5:**
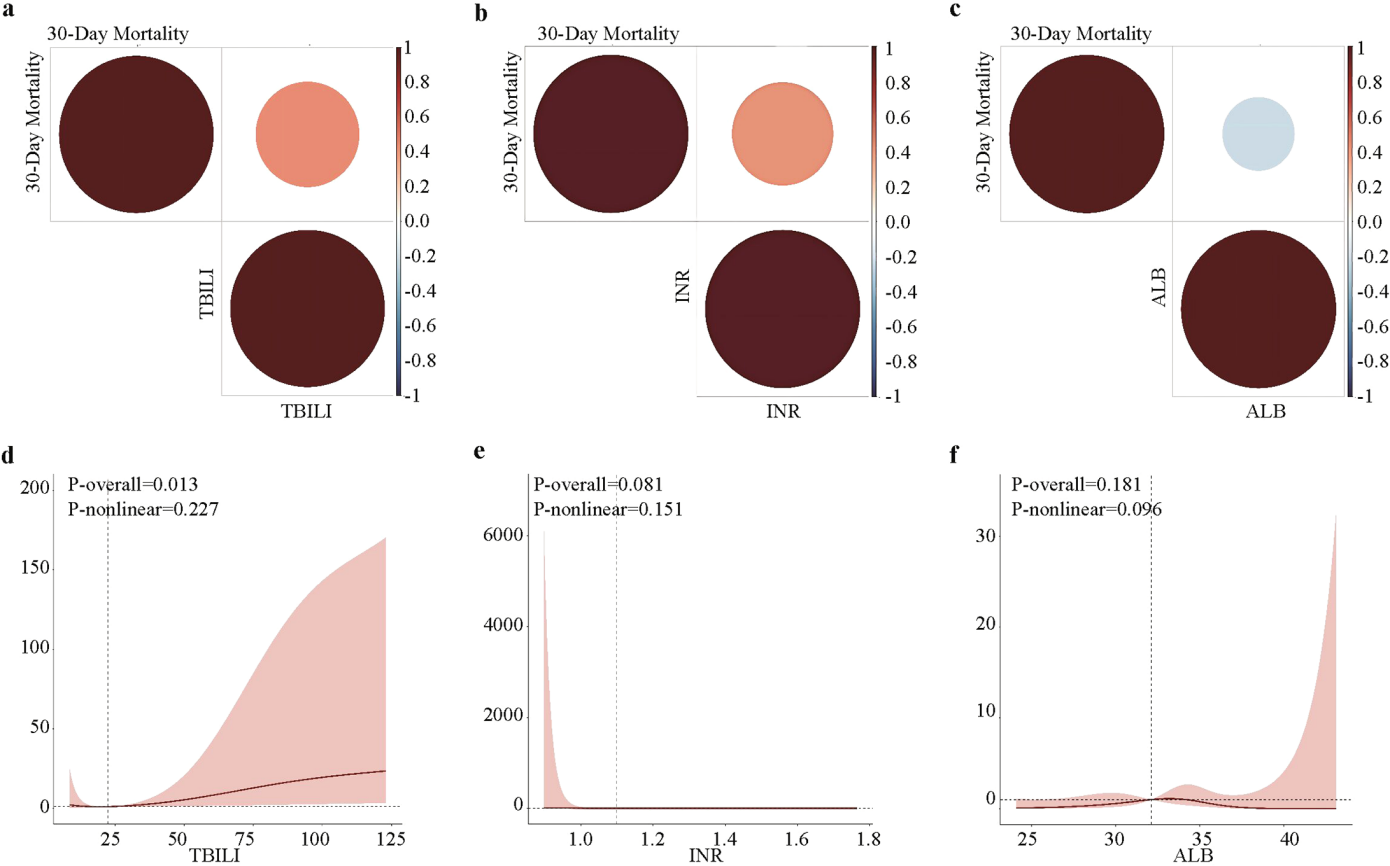
**(a–c)** Analysis of the correlation between TBIL, INR, ALB and 30-day mortality risk; **(d–f)** The association between TBIL, INR, ALB and 30-day mortality risk was shown RCS.

After adjusting for covariates, the analysis showed that TBIL, ALB, and INR all had nonlinear associations with 30-day mortality risk (all P<0.05). For TBIL, risk increased stable when >40 μmol/L(OR = 1.45, 95%CI:1.25-1.68). For ALB, risk increased sharply with decreasing levels when <35 g/L, and leveled off when >35 g/L. INR exhibited a threshold effect: risk increased significantly with elevation when >2.5 (OR = 4.95, 95%CI:3.36-10.11). TBIL, ALB, and INR are important predictors of short-term prognosis in HCC patients, with nonlinear effects (**Figures 5a–c**).

### Construction of a diagnostic nomogram to predict 30-day mortality

**Figure 6:**
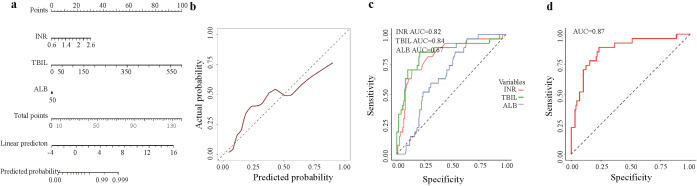
**(a)** Nomogram for the predict of 30-day mortality; **(b)** ROC curves of TBIL, INR, ALB in the HCC rupture population; **(c)** The ROC curve of TBIL and INR in the HCC rupture population; **(d)** The ROC curve of the combination of TBIL and INR in the HCC rupture population.

Logistic regression model was used to analyze the impact of INR, TBIL, and ALB on 30-day mortality risk. The results revealed that INR (OR = 2.10, 95%CI: 1.19-3.71, P = 0.020) and TBIL (OR = 1.03, 95%CI: 1.01-1.04, P = 0.001) were significantly associated with 30-day mortality risk, while ALB (OR = 1.00, 95%CI: 0.90-1.10, P = 0.936) was not significantly associated with 30−day mortality. The model yielded robust results after 100 iterations of Bootstrap sampling validation (**Figures 6a, b**). Further ROC curve analysis demonstrated that among the individual indicators, TBIL exhibited the highest predictive performance (AUC = 0.84, 95%CI: 0.75-0.94), and INR also showed good predictive value (AUC = 0.82, 95%CI: 0.73-0.92), whereas ALB had relatively limited predictive performance (AUC = 0.67, 95%CI: 0.57-0.76)(**Figure 6c**). Subsequently, ROC curve analysis for the combined detection of INR and TBIL showed that the combined index had an AUC of 0.87 (95%CI: 0.79-0.95), indicating that their combination holds significant value for clinical prediction of 30-day mortality risk (**Figure 6d**).

## Discussion

Spontaneous rupture of HCC is a life-threatening severe complication with extremely high short-term mortality. Although previous studies have identified Child-Pugh classification, elevated TBIL, decreased ALB, prolonged international normalized ratio (INR), and ascites as predictive factors for early mortality (12, 13), existing models often rely on single indicators or linear statistical methods, failing to capture complex interactions and nonlinear relationships between variables. This results in limited accuracy in risk stratification, particularly insufficient sensitivity in identifying high-risk patients in emergency settings. In recent years, machine learning models have demonstrated potential in predicting HCC rupture (13, 14).

This study employed a hybrid analytical framework combining machine learning predictive modeling with classical statistical inference to investigate 30-day mortality in patients with ruptured HCC. In Phase 1, among seven machine learning models, the decision tree model demonstrated the highest sensitivity (88.00%) for emergency triage, with TBIL, INR, and ALB identified as the top contributors to model predictions via SHAP analysis. In Phase 2, classical statistical methods confirmed and characterized the nonlinear threshold effects of these predictors: TBIL > 40 μmol/L and INR > 2.5 were associated with significantly elevated mortality risk. The combined detection of TBIL and INR yielded an AUC of 0.87, providing a simple and interpretable tool for rapid clinical risk stratification.

Results of this study showed that, overall, total bilirubin (TBIL) levels were significantly associated with 30-day mortality risk (OR = 1.03; P = 0.001), and International Normalized Ratio (INR) levels were significantly associated with 30-day mortality risk (OR = 2.10; P = 0.020).Further stratified effect analysis revealed a nonlinear association between TBIL and 30-day mortality risk, with a distinct threshold effect: when TBIL exceeded 40 μmol/L, the mortality risk increased significantly (OR = 1.45, 95%CI: 1.25-1.68), indicating a gradual upward trend in mortality risk with increasing TBIL levels and confirming a close association between hyperbilirubinemia and 30-day mortality. Similarly, a nonlinear association was observed between INR and 30-day mortality risk, with an obvious threshold effect: when INR exceeded 2.5, the mortality risk rose significantly (OR = 4.95, P = 0.001).In addition, TBIL exhibited high predictive value for mortality risk (AUC = 0.84, 95%CI: 0.75-0.94), and INR also showed high predictive value for mortality risk (AUC = 0.82, 95%CI: 0.73-0.92). The combined use of INR and TBIL demonstrated extremely high predictive value for 30-day mortality risk in patients with ruptured HCC bleeding (AUC = 0.87, 95%CI: 0.79-0.95).Previous studies have also supported TBIL and INR as independent biomarkers for mortality risk in patients with ruptured HCC, particularly with important implications for predicting short-term and long-term survival rares (11, 16). Survival prediction models for ruptured HCC constructed based on TBIL and INR have also shown high clinical application value (11). It is important to note that the SHAP analysis identifies feature contributions to model predictions rather than establishing causal relationships. The predictive importance of TBIL and INR in our model is, however, consistent with their established pathophysiological roles in liver dysfunction, which have been characterized in prior studies.The biological rationale for the association between TBIL, INR, and short-term mortality in ruptured HCC can be elaborated from the following aspects, based on the existing literature. Firstly, elevated TBIL and INR is a key indicator of hepatic dysfunction, which can lead to reduced synthesis of coagulation factors and increased bleeding risk. TBIL and INR are often used as comprehensive indicators for evaluating liver injury, suggesting that coagulopathy may further exacerbate difficulty in bleeding control after rupture (11, 17). Secondly, ruptured HCC is closely associated with tumor size and progression. Elevated TBIL may reflect a tumor-related systemic inflammatory state, indirectly indicating active tumor growth and increased fragility, leading to uncontrollable bleeding after rupture. Previous studies have shown that the tumor micro-environment plays a critical role in HCC progression, and its components such as hypoxia and inflammatory responses may impair the integrity of the tumor capsule (18, 19). Thirdly, TBIL can serve as a biomarker of oxidative stress and has been confirmed to be associated with mortality risk in liver injury models. Elevated TBIL is often accompanied by lipid accumulation and enhanced oxidative stress responses; reactive oxygen species (ROS) and lipid peroxidation products may damage hepatocytes and vascular endothelial cells, thereby increasing bleeding tendency (20, 21). At last, elevated TBIL levels may restrict clinical treatment choices. In patients with ruptured HCC, although surgical resection can significantly improve long-term survival, patients with high TBIL are often considered surgical contraindications due to insufficient liver function reserve, thereby affecting treatment efficacy and prognosis (22, 23). In summary, in patients with HCC, elevated INR and increased bilirubin levels often co-occur. They act synergistically to amplify mortality risk, collectively indicate the comprehensive state of liver failure, and their combination can identify the “liver failure threshold”. Through synergistic effects, both lead to the complete breakdown of hepatic detoxification, synthetic, and metabolic functions, facilitating life-threatening complications such as hepatic encephalopathy and infection (17, 24). Meanwhile, previous studies have shown that in decision tree models, the sum of the relative contributions of INR and bilirubin exceeds 30%, indicating that they mutually reinforce mortality risk (17). This is also consistent with the findings of our study.

The integration of machine learning and classical statistical methods in this study deserves comment. Machine learning models, particularly decision trees, are well-suited for maximizing predictive sensitivity in complex clinical scenarios, but their “black-box” nature limits direct clinical interpretability. Classical logistic regression, while less flexible in capturing complex interactions, provides transparent effect estimates (odds ratios) and clinically interpretable cutoff values. In this study, the two approaches yielded convergent findings — TBIL and INR emerged as the dominant features in both the SHAP analysis and the logistic regression model — suggesting that these variables are robust predictors regardless of analytical framework. However, we acknowledge that the two-phase approach increases analytical complexity and that future studies should pre-specify the primary analytical framework to avoid potential multiplicity concerns.

Several limitations of this study should be acknowledged. First, the single-center retrospective design with a relatively small sample size (n = 156) may introduce selection bias and limit the generalizability of the findings. Although 5×10-fold cross-validation was employed to internally validate model performance, the lack of an independent external validation cohort precludes definitive conclusions regarding the model’s generalizability. Second, the number of candidate variables (n = 32) is relatively large relative to the sample size, raising concerns about potential overfitting. While the decision tree algorithm inherently performs feature selection — with the final pruned model retaining only 5 effective variables and 8 terminal nodes — the events-per-variable ratio remains modest, and overfitting cannot be fully excluded. Third, although the decision tree model exhibited the highest sensitivity (88.00%), its specificity was relatively low (40.74%), meaning that approximately 60% of actual survivors were classified as high-risk. In the context of emergency triage for a life-threatening condition, we consider this an acceptable trade-off: the primary goal of the model is to minimize missed high-risk patients, and the clinical consequence of false-positive classification is enhanced monitoring rather than invasive overtreatment. Nevertheless, this limitation should be carefully weighed in clinical practice, particularly in resource-constrained settings where unnecessary escalation of care could impose a burden. Fourth, model calibration — i.e., the agreement between predicted probabilities and observed outcomes — was not systematically evaluated. Fifth, the applicability of different models in various clinical scenarios was not compared. Finally, while SHAP analysis suggested a synergistic effect between TBIL and INR, the molecular mechanisms underlying this association were not validated through basic experiments, and the causal relationship requires further verification.

To address these limitations, future studies should focus on: (a) prospective multicenter data collection with standardized protocols to increase sample size and external validity; (b) rigorous model validation using repeated cross-validation, bootstrap resampling, and independent external cohorts; (c) decision curve analysis to quantify the clinical net benefit of the model across different risk thresholds; and (d) in-depth exploration of the biological mechanisms linking TBIL, INR, and short-term mortality in the context of HCC rupture. Nevertheless, for clinicians, it is meaningful to proactively explore the feasibility of preoperative liver function support or staged treatment regimens for high-risk patients identified in this study.

## Data Availability

The raw data supporting the conclusions of this article will be made available by the authors, without undue reservation.

## Supplementary material

Supplementary Table 1 Variance inflation factor (VIF) analysis of candidate variables.

Supplementary Figure 1 **(a)** ROC curves; **(b)** Calibration Curve; **(c)** Decision Curve Analysis, DCA.
