# Supplementary material for: Machine learning prediction of 30-day all-cause mortality risk factors in HCC rupture
Source: Front Oncol. 2026 Jun 23;16:1797271. doi: 10.3389/fonc.2026.1797271 (PMC13337449; doi:10.3389/fonc.2026.1797271)
Supplement: Supplementary Table 1 — Variance inflation factor (VIF) analysis of candidate variables. [file DataSheet1.docx]

**TableS1 Variance inflation factor (VIF) analysis of candidate variables**

| Variable | **VIF** | **TOL** | **Leamer** | **CVIF** | **Klein** | **IND1** | **IND2** |
| --- | --- | --- | --- | --- | --- | --- | --- |
| Age | 1.85 | 0.54 | 0.73 | -1.64 | 0 | 0.12 | 0.98 |
| Sex | 1.47 | 0.68 | 0.82 | -1.31 | 0 | 0.15 | 0.68 |
| Drink | 1.79 | 0.56 | 0.75 | -1.58 | 0 | 0.13 | 0.94 |
| Smoke | 1.66 | 0.60 | 0.78 | -1.47 | 0 | 0.14 | 0.85 |
| Hepatitis | 1.44 | 0.69 | 0.83 | -1.28 | 0 | 0.16 | 0.65 |
| Hypertension | 1.53 | 0.65 | 0.81 | -1.36 | 0 | 0.15 | 0.74 |
| T2DM | 1.59 | 0.63 | 0.79 | -1.41 | 0 | 0.14 | 0.79 |
| Liver Cirrhosis | 1.36 | 0.73 | 0.86 | -1.21 | 0 | 0.17 | 0.57 |
| Ascites | 1.59 | 0.63 | 0.79 | -1.41 | 0 | 0.14 | 0.79 |
| Hypovolemic Shock | 1.47 | 0.68 | 0.82 | -1.30 | 0 | 0.15 | 0.68 |
| INR | 119.03 | 0.01 | 0.09 | -105.54 | 1 | 0.00 | 2.12 |
| APTT | 3.98 | 0.25 | 0.50 | -3.53 | 1 | 0.06 | 1.60 |
| TT | 2.31 | 0.43 | 0.66 | -2.05 | 1 | 0.10 | 1.21 |
| PT | 120.16 | 0.01 | 0.09 | -106.54 | 1 | 0.00 | 2.12 |
| PLT | 1.43 | 0.70 | 0.84 | -1.27 | 0 | 0.16 | 0.64 |
| HGB | 1.80 | 0.56 | 0.75 | -1.59 | 0 | 0.13 | 0.95 |
| ALB | 2.32 | 0.43 | 0.66 | -2.05 | 1 | 0.10 | 1.21 |
| Cr | 1.47 | 0.68 | 0.83 | -1.30 | 0 | 0.16 | 0.68 |
| AST | 1.67 | 0.60 | 0.77 | -1.48 | 0 | 0.14 | 0.86 |
| TBILI | 2.16 | 0.46 | 0.68 | -1.91 | 1 | 0.11 | 1.14 |
| D-dimer | 2.67 | 0.37 | 0.61 | -2.37 | 1 | 0.09 | 1.33 |
| Child-Pugh B | 2.65 | 0.38 | 0.61 | -2.35 | 1 | 0.09 | 1.33 |
| Child-Pugh C | 4.22 | 0.24 | 0.49 | -3.74 | 1 | 0.05 | 1.63 |
| Single Tumor | 1.36 | 0.74 | 0.86 | -1.20 | 0 | 0.17 | 0.56 |
| Multiple Tumor | 1.15 | 0.87 | 0.93 | -1.02 | 0 | 0.20 | 0.28 |
| Maximum Tumor Diameter | 1.62 | 0.62 | 0.79 | -1.44 | 0 | 0.14 | 0.82 |
| PVTT | 2.09 | 0.48 | 0.69 | -1.86 | 1 | 0.11 | 1.11 |
| **Extrahepatic Metastasis** | 1.36 | 0.74 | 0.86 | -1.20 | 0 | 0.17 | 0.56 |
| BCLC stage | 2.23 | 0.45 | 0.67 | -1.97 | 1 | 0.10 | 1.18 |

Abbreviations:INR:international normalized ratio; ALT: glutamic-pyruvic transaminase; AST: glutamic oxalacetic transaminase; APTT:activated partial thromboplastin time; TT: thromboplastin time;PT:prothrombin time;HGB:hemoglobin;Alb:albumin;AFP:alpha fetal protein; PVTT：portal vein tumor thrombus；BCLC:Barcelona Clinic Liver Cancer.
