# Supplementary figures and images for: Machine learning prediction of 30-day all-cause mortality risk factors in HCC rupture
Source: Front Oncol. 2026 Jun 23;16:1797271. doi: 10.3389/fonc.2026.1797271 (PMC13337449; doi:10.3389/fonc.2026.1797271)

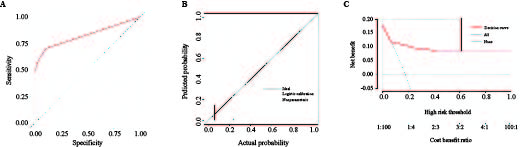

Supplement: Supplementary Figure 1 — (a) ROC curves; (b) Calibration Curve; (c) Decision Curve Analysis, DCA. [file Image1.jpeg]
